# An equitable, safe, and reliable blood supply in the Western Pacific

**DOI:** 10.1016/j.lanwpc.2025.101647

**Published:** 2025-07-31

**Authors:** The Lancet Regional Health – Western Pacific

“Give blood, give hope: together we save lives” is the theme for World Blood Donor Day 2025. This day honours the contributions of blood donors whose generosity is vital to the many people who rely on blood transfusions for their life and health. One unit of safe blood can save three people's lives. The Western Pacific region has one of the highest proportions of countries reporting fewer than ten donations per 1000 people globally. Although an additional 4.15 million donations from voluntary unpaid donors were reported between 2008 and 2018 in the region, this figure remains far below the WHO-recommended goal of ten donations per 1000 people needed to meet the health-care demands of the population.

The Western Pacific region bears a substantial burden of health inequities related to conditions and settings that could be exacerbated by shortages in safe and reliable blood supply. For example, thalassaemia is one of the most common inherited disorders in Asia. Additionally, the Pacific Island Countries and Territories experience higher rates of maternal and perinatal morbidity and mortality, with services frequently constrained by limited resources and health-care workforce. In low-income countries worldwide, children under the age of 5 years receive 54% of blood transfusions. Blood donation rates in low-income and middle-income countries (LMICs) continue to lag behind those in high-income countries, with a median of 6.6 donations per 1000 people compared with 31.5, respectively. Studies on blood donors and donation practices across the region remain scarce, hindering efforts to develop tailored initiatives that ensure equitable and reliable access to safe blood. Existing evidence suggests significant variation in donor characteristics. In South Korea, most donors tend to be younger (under 30 years); whereas in Japan, most donors are aged 50 years and older. Most donors in Japan, South Korea, Singapore, Taiwan, Chandigarh (India), and Yogyakarta (Indonesia) were male, not including Mongolia where a higher proportion of donors were female (62.6%).

Addressing disparities related to gender, age, socioeconomic status, and ethnicity as well as social and cultural factors in the donation processes is necessary for fostering an equitable and diverse donor base. This is especially important given demographic shifts in the region, the increasing burden of non-communicable diseases, and climate-related health challenges that could further compromise the blood donation process and sustainability of blood supplies, ultimately widening the gap between need and supply. Data from ten blood collection agencies across Asia between 2017 and 2020, including over 9.5 million donations, reveal variability in donor eligibility criteria and practices. Most agencies used male haemoglobin thresholds lower than the WHO anaemia cutoff of 13.0 g/dL and accepted donations from individuals weighing less than 50 kg. In Hong Kong and Taiwan, individuals who were deferred from donating blood were mostly females and had lower bodyweight and estimated blood volume. This study highlights the need for further research into haemoglobin reference values and blood volume estimation formulas tailored to the region's diverse populations. Additionally, routinely collecting data such as weight and height is vital for more accurate blood volume estimates and for safeguarding donor health. In Asian countries, reimbursement (eg, meal and transport compensation) for blood donations might still be common, further highlighting the need to improve education and donation processes to ensure safety and encourage repeat donations from voluntary unpaid donors.

The higher prevalence of transfusion-transmissible infections such as HIV, hepatitis B and C, and syphilis in LMICs further contributes to fewer donations from lower-risk groups. Although evidence on how donor exclusion criteria impact blood supply remains limited in the region, it is crucial to ensure that screening processes reduce stigma and discrimination against donors from minority or vulnerable groups to promote an equitable and diverse donor base. In 2021, a historic change to blood donation eligibility rules in the UK meant that a greater number of men who had sex with men were able to donate blood. This change shifted the focus to assessing risk based on individual health-related characteristics and behaviours, a significant step toward more equitable donation practices. A 2019 white paper from the Asia Pacific Blood Network discussed risk reduction strategies for dengue, proposing that implementing screening tests or pathogen reduction methods in countries with high dengue prevalence, along with targeted testing of donors in outbreak areas within low-prevalence regions, could be more cost-effective and help minimise negative impacts on potential donors and blood availability.

Considering the geographical diversity in the Western Pacific region, including remote islands and rural regions, innovative and adaptable approaches to the collection, storage, processing, and transportation of blood products are essential. For example, the Blood DESERT (Blood Delivery via Emerging Strategies for Emergency Remote Transfusion Coalition) approach aims to identify blood deserts, defined as “geographical regions where essential clinical demand for blood components cannot be met at the point of care in a timely and affordable manner, in at least 75% of cases where transfusion is needed”. Accordingly, this approach could help map communities at greatest risk of morbidity and mortality due to blood shortages, and inform policy development, public health awareness campaigns, and guide investments in blood donation infrastructure in the region.

The Western Pacific region has made important progress in improving blood safety and availability. Continued efforts focused to improve research, donation practice and safety, infrastructure, community awareness, and regulatory policies are essential to achieving a future where safe, equitable, and sustainable blood supply are available to all in the region.

